# Supplementary figures and images for: Evolutionary dynamics of microRNA target sites across vertebrate evolution
Source: PLoS Genet. 2020 Feb 3;16(2):e1008285. doi: 10.1371/journal.pgen.1008285 (PMC7018135; doi:10.1371/journal.pgen.1008285)

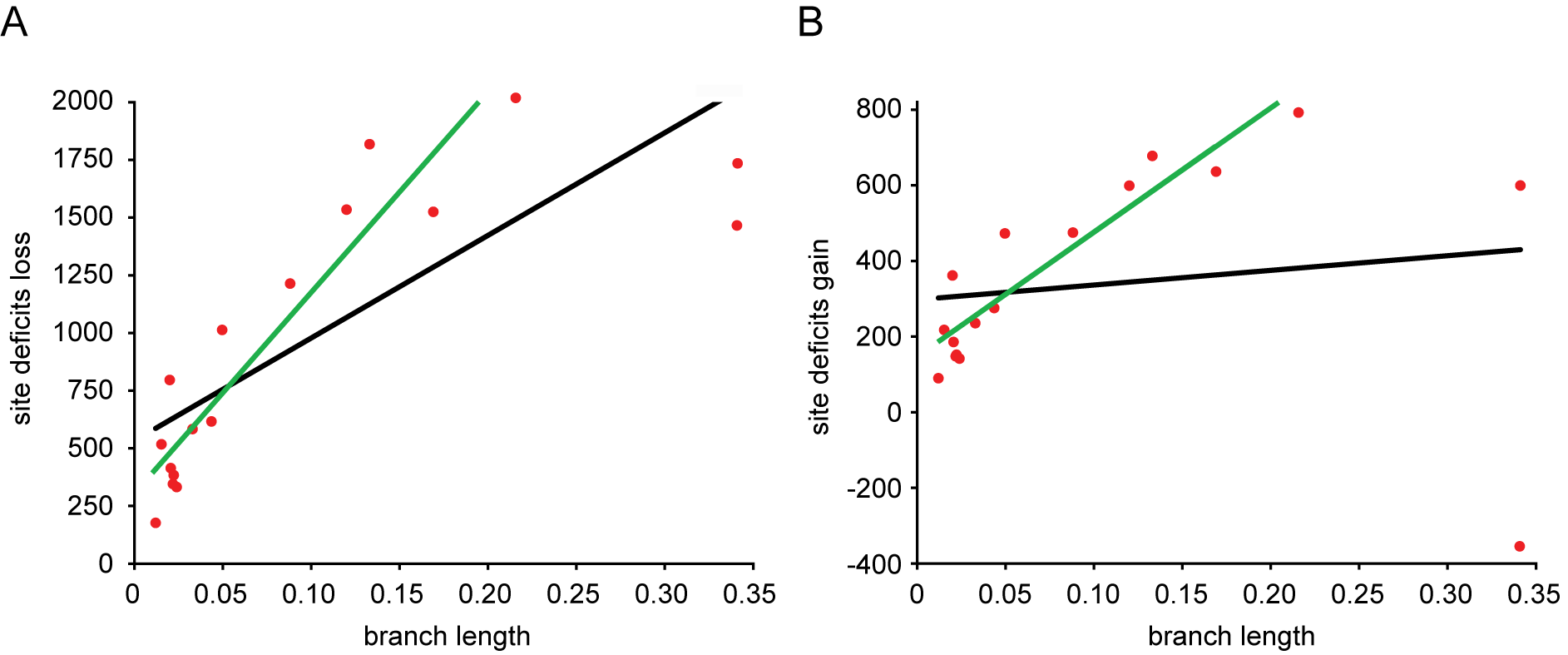

Supplement: S1 Fig — We carried out the analyses of the main text for individual branches along the mammalian phylogeny. When analyzing the 15 shortest branches, in green (as measured by depletion of observed miRNA target site turnover events relative to expected events, see bottom portion of S1 and S2 Tables), we observed R2 values of 0.883 (P = 2.0x10^-7^) and 0.865 (P = 5.1x10^-7^) for losses (A) and gains (B) respectively. When two very long branches were included (black lines) we observed R2 values of 0.64 (P = 1.1x10^-4^) and 0.022 (P = 0.567) for losses (A) and gains (B) respectively. (TIF) [file pgen.1008285.s001.tif]

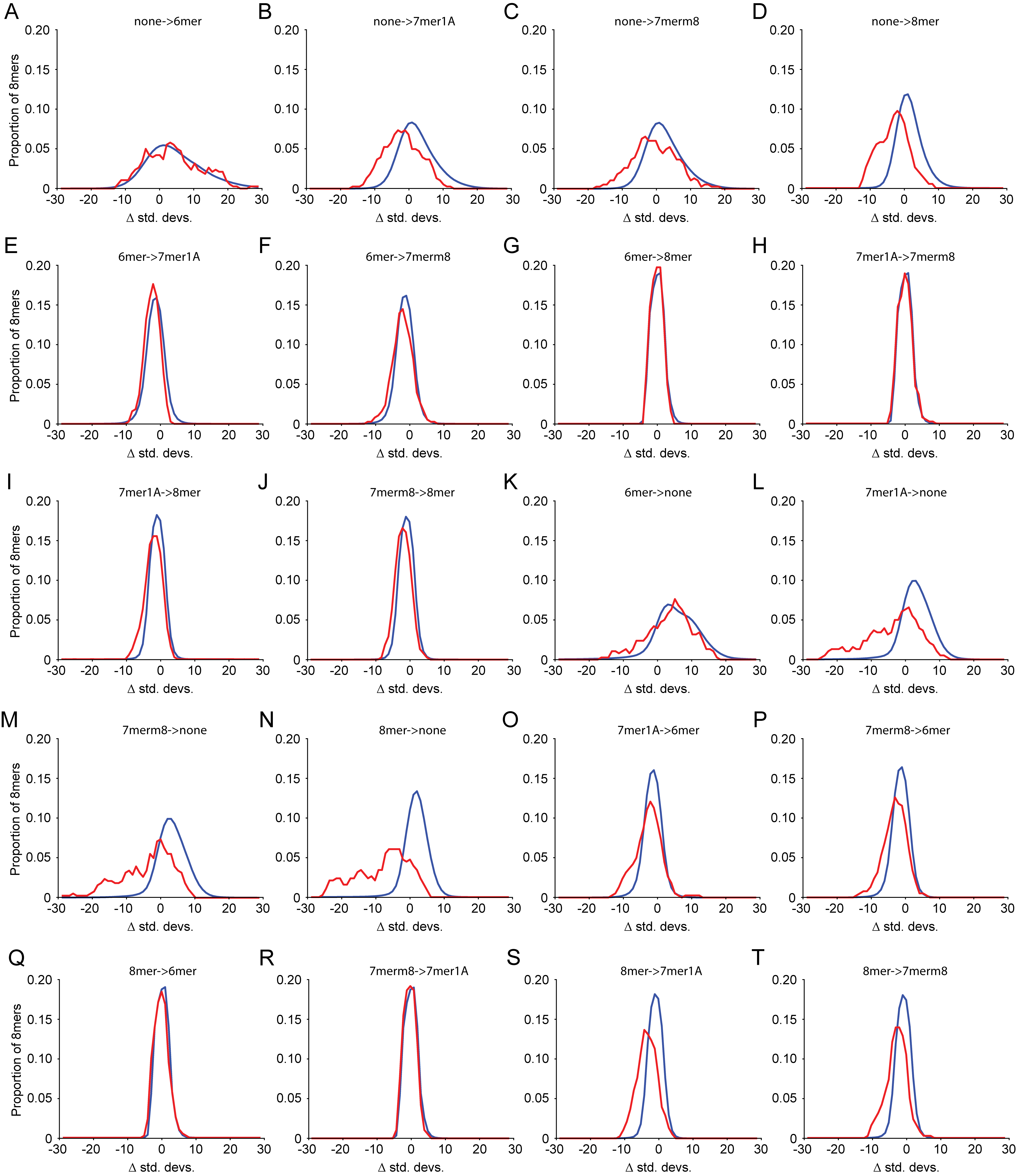

Supplement: S2 Fig — (A-T) Analysis of standard deviation metrics, calculated as in Fig 2, for target site interconversion events between the site types none, 6mer, 7merA1, 7merm8, and 8mer. Wilcoxon rank sum statistics for miRNAs (red line) relative to all 8mers (blue line) are captured as p-values and shown in Fig 7. (TIF) [file pgen.1008285.s002.tif]

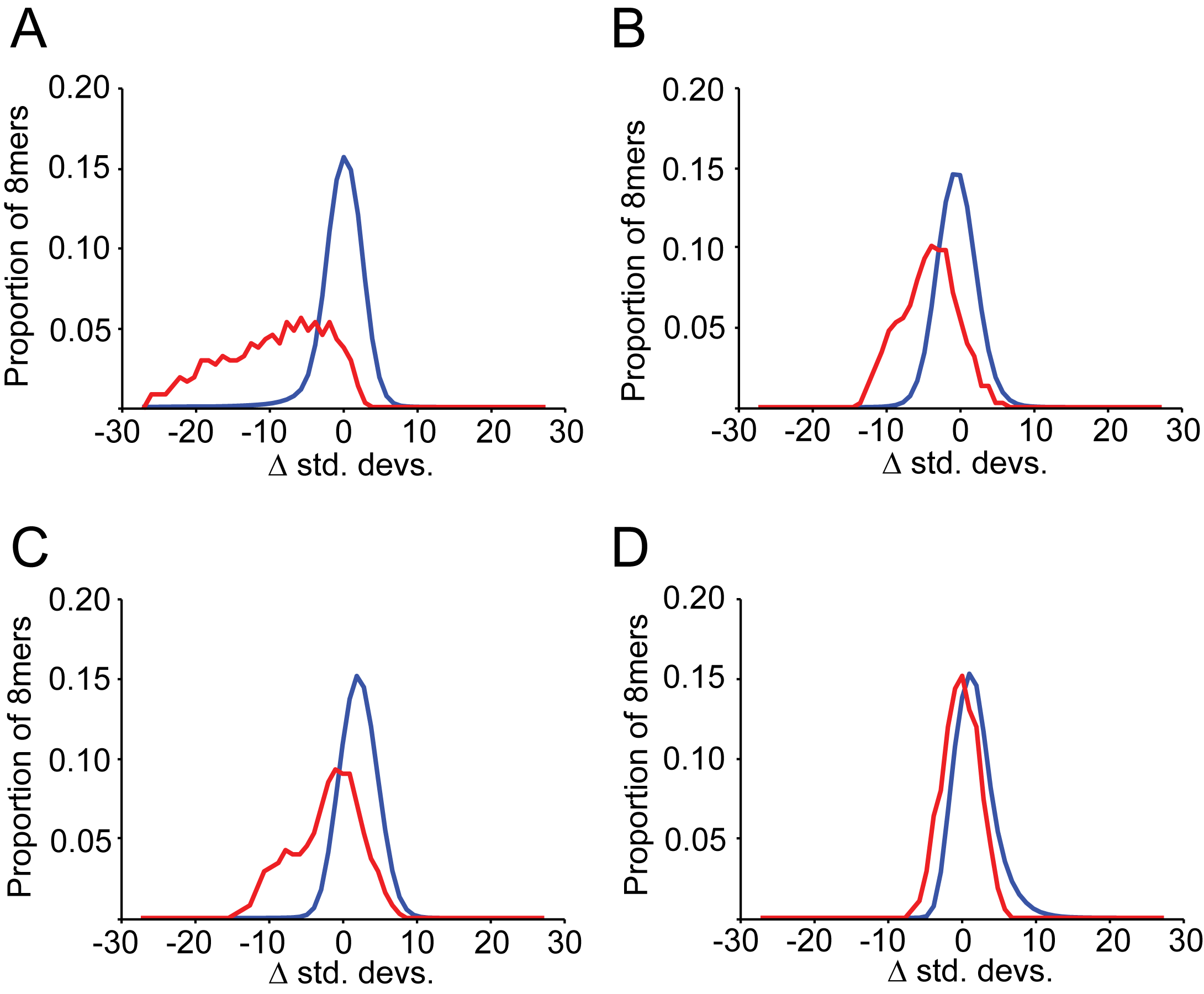

Supplement: S3 Fig — (A, B) AU-rich regions of 3′UTRs analyzed with respect to miRNA target site losses (A) and miRNA target site gains (B). (C,D) AU-poor regions of 3′UTRs analyzed with respect to miRNA target site losses (C) and miRNA target site gains (D). (TIF) [file pgen.1008285.s003.tif]

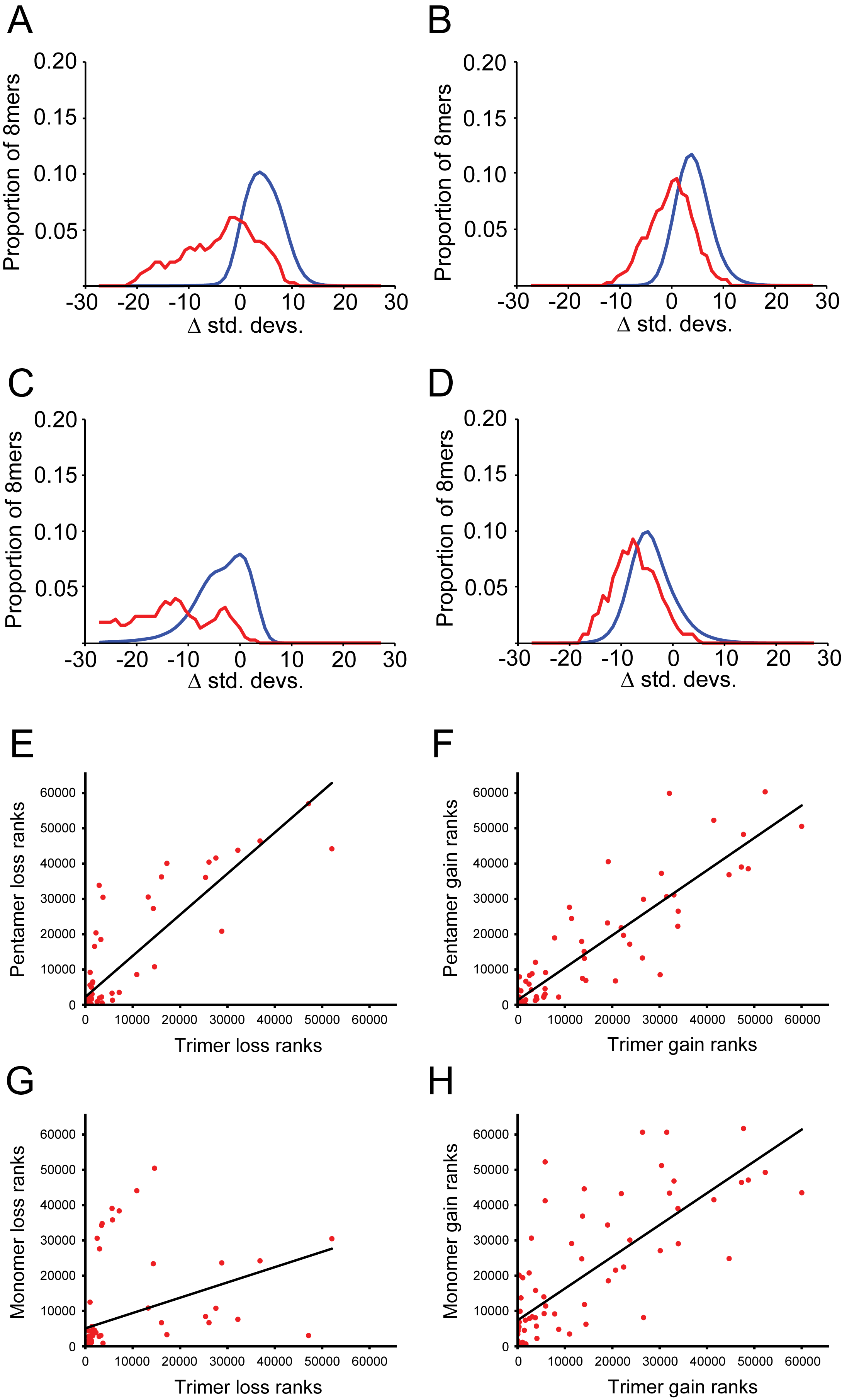

Supplement: S4 Fig — The flanking single nucleotide model described in the text (‘trimer’ model) was compared to a model that utilized two flanking nucleotides on each side (A, B), a ‘pentamer’ model, and to (C, D), a model that measured frequencies of nucleotide substitution without considering flanking nucleotides on either side (a ‘monomer’ model). Analysis performed as described in Fig 2A and 2B (losses on the left, gains on the right). (E, F) Correlations between miRNA target site loss (E) and gain (F) ranks between the ‘trimer’ (x-axis) and ‘pentamer’ model (y-axis). (G, H) Correlations between the ‘trimer’ (x-axis) and ‘monomer’ (y-axis) models, for losses (G) and gains (H), respectively. (TIF) [file pgen.1008285.s004.tif]
